# Effects of polyaluminum chloride (PAX-18) on the relationship between predatory fungi and *Lecane* rotifers

**DOI:** 10.1007/s11356-021-16952-2

**Published:** 2021-10-21

**Authors:** Edyta Fiałkowska, Wojciech Fiałkowski, Christopher G. Wilson, Agnieszka Pajdak-Stós

**Affiliations:** 1 Institute of Environmental Sciences, Faculty of Biology, Jagiellonian University, Gronostajowa 7, 30-387 Kraków, Poland; 2 Department of Zoology, University of Oxford, 11a Mansfield Road, Oxford, OX1 3SZ UK

**Keywords:** *Zoophagus* sp., *Lecophagus* sp., Conidia, Fungi mycelium, Wastewater treatment, Inorganic coagulants

## Abstract

PAX-18 (polyaluminum chloride) is frequently used in WWTPs (wastewater treatment plants) to overcome sludge bulking. An alternative biological method is the usage of *Lecane* rotifers, which can be endangered by predacious fungi. We investigated the influence of different PAX-18 concentrations on the relationship between *Lecane inermis* and predacious fungi (*Zoophagus* and *Lecophagus*) differing in feeding mode. High PAX concentration (6 mg Al^3+^ L^−1^) strongly limited the number of the rotifers, which in low concentration (1.2 mg Al^3+^ L^−1^), after an initial decline, increased, but significantly slower than in control. Under the simultaneous influence of *Lecophagus* and PAX, rotifers were driven almost extinct at the high concentration, but survived at the lower concentration and increased in the control. When treated with *Zoophagus*, only one or two rotifers survived in treatments and control. High concentrations of PAX significantly restricted the growth of fungi, whereas in low concentrations and control conditions, their length increased, with *Zoophagus* growing much quicker than *Lecophagus*. *Zoophagus* was significantly more efficient in trapping rotifers regardless of PAX concentration. The trapping ability of mycelium following extended exposure to PAX was strongly limited at high concentrations, in comparison to control. Conidia of *Zoophagus* turned out to be considerably more resistant to PAX-18 and starvation than *Lecophagus* conidia.

**Supplementary Information:**

The online version contains supplementary material available at 10.1007/s11356-021-16952-2.

## Introduction

The activated sludge process is the most common method of wastewater treatment all over the world. In properly functioning activated sludge, flocs consisting of different functional groups of bacteria, exopolymeric substances, protozoans, and metazoans easily settle in the final clarifier, and purified water could be effectively separated from the sludge. If the balance between different constituents of the activated sludge community is disrupted due to changes in temperature, type of sewage, or operational problems, the sedimentation process could be hindered. There are different types of malfunctions resulting in solid separation problems. Among them are as follows: dispersed growth of microorganisms when they do not stick together, formation of pinpoint flocs (PP) when only very small, and mechanically fragile flocs are present; and zoogleal bulking caused by excessive amount of extracellular material (EPS) produced by microorganisms (Jenkins et al. 2003; Mesquita et al. 2011). However, the most common problem of liquid–solid separation is activated sludge bulking caused by an overproliferation of filamentous bacteria (Eikelboom 2000; Jenkins et al. 2003). To limit the development of such bacteria, WWTPs operators use various coagulants such as ferrous chloride, ferric chloride, hydrated aluminum sulfate, polyaluminum chloride, or cationic polymers (Jenkins et al. 2003; Mamais et al. 2011). However, so far, no method is 100% efficient. In the case of the commonly occurring filamentous bacterium *Microthrix parvicella*, one of the most effective are coagulants such as polyaluminum chloride PAX (Roels et al. 2002; Mamais et al. 2011; Pal et al. 2014). This method, however, has its drawbacks. Although aluminum is abundant in the environment, it is toxic at high concentrations. Havas (1985) found that Al^3+^ was toxic to the cladoceran *Daphnia magna* at 0.32 mg Al^3+^ L^−1^ (pH 6.5). Experiments by Jancula and co-workers showed that EC50 values for *D. magna* fluctuate between 9.89 and 54.29 mg Al^3+^ L^−1^ (Jančula et al. 2011). In experiments on macroinvertebrates *Hyalella azteca* (Crustacea), *Gyraulus* sp. (Gastropoda), *Paratanytarsus* sp., and *Zavrelimyia* sp. (Diptera), Havens (1993) showed higher mortality at pH 4.5 with the addition of 0.2 mg Al^3+^ L^−1^ than with pH 4.5 alone. Numerous researchers also demonstrated the harmful effect of aluminum on human health, pointing to the use of aluminum-based coagulants applied in the treatment of drinking water as well as wastewater as a potential source of aluminum in potable water (Crisponi et al. 2013 and literature therein; Frisbie et al. 2015).

The application of polyaluminum chloride, apart from being expensive and introducing huge amounts of aluminum into the environment, is not always effective (Drzewicki 2009). Another, more environmentally friendly method proposed for controlling filamentous bacteria is the application of rotifers. The species *Lecane inermis* is capable of ingesting not only *M. parvicella*, but also other types of filamentous bacteria such as *Nostocoida limicola*, type 021 N, type 0092, *Thiothrix*, *Haliscomenobacter hydrosis*, and Actinomycetes (Fiałkowska and Pajdak-Stós 2008; Pajdak-Stós and Fiałkowska 2012; Kocerba-Soroka et al. 2013; Kowalska et al. 2014, 2016; Drzewicki et al. 2015; Pajdak-Stós et al. 2017a, b).

Our earlier observations revealed that rotifers used as a biological tool for preventing bulking are potentially endangered by fungi preying on them (Pajdak-Stós et al. 2016). Although predatory fungi feeding on rotifers were reported from natural environments (Sommerstorff 1911; Barron et al. 1990; Glockling 1997; Tanabe et al. 1999; McInnes 2003), until recently, reports on their occurrence in WWTPs were scarce (Cooke and Ludzack 1958; Pipes and Jenkins 1965; Sladka and Ottova 1973). In the last few years, however, it turned out that predatory fungi trapping rotifers are quite common in full-scale WWTPs (Fiałkowska et al. 2016) and that they could be a real danger for rotifer populations inhabiting activated sludge (Pajdak-Stós et al. 2016; Fiałkowska and Pajdak-Stós 2018).

In the preliminary experiments in which we investigated the influence of rotifers and PAX on filamentous bacteria in activated sludge, we noticed an interesting pattern. The predatory fungus did not appear in any of eight experimental vessels where PAX was applied, whereas it developed in seven out of eight control vessels. These observations suggested that PAX might strongly limit the growth of the fungi.

Given that a predatory fungus could be a real threat for beneficial rotifers, we aimed to test how PAX affects two distinct genera of fungi feeding on rotifers: *Lecophagus* and *Zoophagus*, when applied at aluminum ion concentrations recommended for bulking control. As it was shown that rotifers such as *L. inermis* are vulnerable to aluminum ions present in simple salts (Klimek et al. 2013), we also decided to check if the application of PAX in a concentration lower than recommended for bulking control could have an indirect positive effect on rotifers in activated sludge by suppressing predatory fungi.

The main goal of our investigation was to test a hypothesis that PAX has a stronger negative impact on predatory fungi than on rotifers, thus lessening the pressure predatory fungi exert on the *Lecane* rotifers. As a result, the effectiveness of *Lecane* in reducing the number of the filamentous microorganisms responsible for sludge-bulking would not be compromised.

Elucidating the impact of PAX coagulant on activated sludge microorganisms and their ecological relationships will help to select and optimize the method of reducing sludge bulking, aiming to be effective on the one hand, and environmentally friendly on the other.

## Material and methods

For all experiments, the clone 1.A2.15 of *Lecane inermis* (Bryce, 1892) was used. It was obtained from a single individual isolated from an activated sludge sample, maintained in Petri dishes in Żywiec® spring water in darkness, at 20 °C (Fiałkowska et al. 2020), and fed with nutrition powder NOVO (patent EP2993978(A1)) (Pajdak-Stós et al. 2017a).

The experiments were conducted on two strains of predatory fungi belonging to different genera differing in the mode of feeding. *Zoophagus* belongs to the order Zoopagales which contains obligate parasites and predators (Corsaro et al. 2018; Davis et al. 2019) and cannot be maintained in pure culture. *Lecophagus*, belonging to the Orbiliales, preys on rotifers, tardigrades, and nematodes but is also able to utilize nutrients dissolved in the medium and thus can be maintained in pure culture.

*Lecophagus* sp. strain Z1 originates from a wastewater treatment plant located in southern Poland, as detailed by Fiałkowska and Pajdak-Stós (2018). The strain was isolated to pure culture on potato dextrose agar using the methods of Barron (2004). It has been deposited in the USDA Agricultural Research Service Collection of Entomopathogenic Fungi (ARSEF), under accession code ARSEF 14203.

*Zoophagus* sp. strain Tk1 originates from another wastewater treatment plant in southern Poland (Pajdak-Stós et al. 2016). Due to the inability to obtain pure culture, the fungus is not deposited in any collection, but the cultures are constantly maintained in test plates in Żywiec® spring water in darkness, at 20 °C, and fed with *Lecane inermis* (clone 1.A2.15) once per 3 weeks. All the cultures are constantly maintained in the laboratory of Aquatic Ecosystems Group, Institute of Environmental Sciences, Jagiellonian University in Kraków, Poland.

To check the identity of each fungal strain, genomic DNA was extracted from freshly harvested rotifer-trapping mycelium by the CTAB method of Sung et al. (2001). An approximately 1 kilobase section of the gene encoding the small ribosomal subunit (nrSSU) was amplified by PCR, using the primers NS1 and NS4 (White et al. 1990). The reagents (GE Healthcare Illustra™ PuReTaq Ready-To-Go™ PCR Beads) were dissolved in 20 µL of MilliQ water, with 2 µL of each primer (at 10 µM) and 1 µL of extracted DNA (~ 5 ng) in TE buffer for a total volume of 25 µL. Reactions were performed on an Eppendorf Mastercycler Gradient with initial denaturation at 94 °C for 4 min, then 35 cycles (comprising 94 °C denaturation for 40 s, 51.5 °C annealing for 30 s, and 72.5 °C extension for 1 min), and then final extension at 72.5 °C for 5 min.

PCR products were purified using an Illustra™ GFX PCR DNA and Gel Band Purification kit (GE Healthcare, Little Chalfont, UK) and Sanger-sequenced directly and bidirectionally by Macrogen Europe (Amsterdam, the Netherlands) using an ABI 3730xl DNA Analyzer (Applied Biosystems), with the amplification primers and a supplementary internal primer for *Zoophagus* (ZSU990R, 5′-GGTATCTGATCGTCTTTGA-3′). Chromatograms were aligned, visually checked, and trimmed using Geneious® v. 8.1 (Biomatters Ltd., Auckland, New Zealand) and the assembled sequences (1051 bp for *Lecophagus* and 956 bp for *Zoophagus*) were deposited at NCBI GenBank (accession codes MZ326345 and OK156471 respectively; Supplementary File 1).

Each nrSSU sequence was queried against the NCBI nucleotide database (nt) using blast with standard settings (https://blast.ncbi.nlm.nih.gov/). For *Lecophagus* sp. Z1 (MZ326345), the highest scoring hit (99.4% pairwise identity) was *Lecophagus muscicola* (GenBank AB001108), sequenced by Tanabe et al. (1999). For *Zoophagus* sp. Tk1 (OK156471), the highest scoring hit (91.6% pairwise identity) was *Zoophagus insidians* (AB016009), sequenced by Tanabe et al. (2000).

In the experiments, polyaluminum chloride (PAX-18) (Kemipol Sp. z o. o. Police, Poland) was used. The coagulant contains 9.0 ± 0.2% of aluminum; its pH is 0.9 ± 0.3 and density 1.35–1.37 g/cm^3^. The concentration of aluminum ions recommended by the manufacturer for WWTPs is significantly higher than that applied by operators to control bulking, mainly due to the high cost of PAX-18 (personal communication). Actual concentrations of PAX applied range between 0.6 and 3.0 g AL^3+^ kg^−1^ MLSS (mixed liquor suspended solids) respectively.

To obtain solutions containing 1.2 mg L^−1^ and 6 mg L^−1^ aluminum ions, concentrations effective in the range of MLSS values between 2 and 5.5 g L^−1^, 10 µL, and 50 µL of PAX-18 respectively were diluted in 1 L of deionized water. Because the addition of PAX in the concentration of 6 mg Al^3+^ L^−1^ decreases pH to 6.4, 2.5% NaOH solution was added to obtain PAX solution with pH = 7.0 (hereafter called normalized). From now on, these solutions will be referred to as PAX 1.2, PAX 6 pH 6.4, and PAX 6 pH 7 respectively.

### Experiment I: the effect of PAX on Lecane rotifers in the absence of fungi

Twenty individuals of *L. inermis* were transferred with a Pasteur pipette into each well of a 24-well culture test plate (TPP). Each well was filled with 1.5 mL of medium and 30 µL of 3‰ molasses solution (Greenland Technologia, Poland) as a food source for rotifers. Then, the 24 wells were divided into 4 experimental groups: [1] control; [2] PAX 1.2; [3] PAX 6 pH 7; and [4] PAX 6 pH 6.4—each with 6 replicates, where: control, Żywiec® spring water; PAX 1.2, PAX solution containing 1.2 mg L^−1^ of aluminum ions; PAX 6 pH 7, PAX solution containing 6 mg L^−1^ of aluminum ions normalized to pH 7.0; and PAX 6 pH 6.4, PAX solution containing 6 mg L^−1^ of aluminum ions without pH normalization.

The experimental plate was maintained in darkness at 20 °C. On the 1st, 4th, and 7th days, after the beginning of the experiment, the number of active rotifers was counted using an inverted microscope (Olympus IX 71).

### Experiment II: the influence of PAX on interactions between early developmental stages of predatory fungi and Lecane rotifers

To check the simultaneous influence of PAX and the presence of predatory fungus on *L. inermis*, we prepared experimental wells according to the protocol described above for testing the effect of PAX on the rotifers. Each well was filled with 1.5 mL of medium and 30 µL of 3‰ molasses solution (Greenland Technologia, Poland) as a food source for rotifers; then PAX treatments were applied as described above. Additionally, each well of the test plate was inoculated with 3–5 *Lecophagus* conidia (“L”).

In this way, four experimental groups were obtained: [1] Żywiec + L (control); [2] PAX 1.2 + L; [3] PAX 6 pH 7 + L; and [4] PAX 6 pH 6.4 + L—each with 6 replicates. On the 4th and 7th days, the length of the mycelium growing out of the initial conidia was measured under an inverted microscope Olympus IX 71 equipped with a digital camera PixelLink and image analysis program (NIS Elements 3.0). A corresponding experiment was conducted on *Zoophagus* conidia (“Z”), with minor adjustments concerning the timing of mycelium measurements and with the following experimental groups: [1] Żywiec + Z (control); [2] PAX 1.2 + Z; [3] PAX 6 pH 7 + Z; and [4] PAX 6 pH 6.4 + Z—each with 6 replicates.

The experiments were maintained in darkness at 20 °C. Along with measuring mycelium, the number of active (moving) rotifers was recorded on the 1st, 4th, and 7th day since the beginning of the experiment. Additionally, in the experiment with *Lecophagus*, on the 4th day, we noted the wells in which a second generation of conidia appeared.

In the case of *Lecophagus*, the mean length of mycelium per initial conidium is calculated and used to calculate a mean growth rate, according to the formula: *r* = 1/*t* (*ln*(*N*_t_) − *ln*(*N*_0_)) where *t* is time (days) between measurements, *N*_t_ is the length of mycelium on the 7th day, and *N*_0_ is the length of mycelium on the 4th day from the beginning of the experiment. Since *Zoophagus* mycelium grew much faster than that of *Lecophagus*, in the case of the former, the total length of mycelium per well was measured on the 4th day of the experiment except for control wells in which the length already exceeded 20 000 µm. It was impossible to measure *Zoophagus* mycelium precisely on the 7th day, because long fragments protruded upwards from the bottom of the well into the overlying medium, so the total length of *Zoophagus* mycelium was only measured on the 4th day, and the growth rate for this fungus was not calculated.

### Experiment III: the effect of PAX on the trapping efficiency of developed mycelium

To eliminate a potential indirect effect of PAX on the fungus, caused by changing availability of rotifer prey, an experiment testing the direct effect of PAX solution on developed mycelium was conducted.

Conidia of *Lecophagus* (approximately 10 per well) were transferred with the Pasteur pipette into 18 wells of 24-well culture test plates. Into each well, 25 µL of dense rotifer culture was added at the start and on the 4th day to ensure unlimited access to food for the predatory fungus. Wells were filled with 1.5 mL of Żywiec® spring water and maintained in darkness at 20 °C. After 8 days, when mycelium was already well developed, the wells were divided into three experimental groups with six replicates per group. The medium was carefully removed and replaced with the 1.5 mL/well of following solutions: Żywiec®; PAX 1.2; PAX 6,  pH = 6.4.

To test how PAX affected the *Lecophagus* mycelium, after the following 10 days, the medium was carefully removed, and the wells were twice delicately rinsed with Żywiec® spring water. Then, 1.5 mL of Żywiec® enriched with 30 µL of molasses solution along with about 100 rotifers was added into each well. Three hours later, rotifers trapped by the fungus were counted. On the next day, all active rotifers were counted in each well.

This procedure was repeated in the case of *Zoophagus* mycelium except that the time for development of mycelium was limited to 4 days since the previous experiment showed that *Zoophagus* grows distinctly faster than *Lecophagus*.

### Experiment IV: conidia resistance to PAX and starvation

A final experiment was designed to check if conidia, often considered more resistant to harsh environmental conditions than mycelium, can survive even higher PAX concentrations. The experiment was conducted in three 24-well test plates. Each plate contained 18 experimental wells. Into each well, 10 µL of a well-mixed dense suspension of *Lecophagus* conidia (approximately 1200 conidia/mL) were transferred; then the wells were divided into three experimental groups as follows: [1] control, conidia in 1.5 mL of Żywiec® spring water; [2] PAX 6, conidia in PAX solution with 6 mg L^−1^ of aluminum ions; and [3] PAX 12, conidia in PAX solution with 12 mg L^−1^ of aluminum ions.

At intervals of 3 weeks, 1 month, and 2 months (using independent wells), conidia was twice carefully rinsed with Żywiec® spring water and then provided with approximately 270 rotifers per well. After 3 h, all rotifers trapped by conidia were counted, and the average number of caught rotifers per conidium was calculated. The experiment was repeated for *Zoophagus* conidia according to the same procedure.

The results obtained from the experiments were found to be normally distributed. Therefore, we decided to use a parametric test and analyze the results by means of factorial ANOVA, supplemented by Tukey HSD post hoc analysis, to reveal significant differences between the reactions of the investigated fungi, as well as their prey, to different concentrations of aluminum ions. Statistical analyses were performed using the GLM module of the STATISTICA package v. 12.5 (StatSoft, Inc. 2014).

## Results

### Experiment I: the effect of PAX on Lecane rotifers in the absence of fungi

**Fig. 1:**
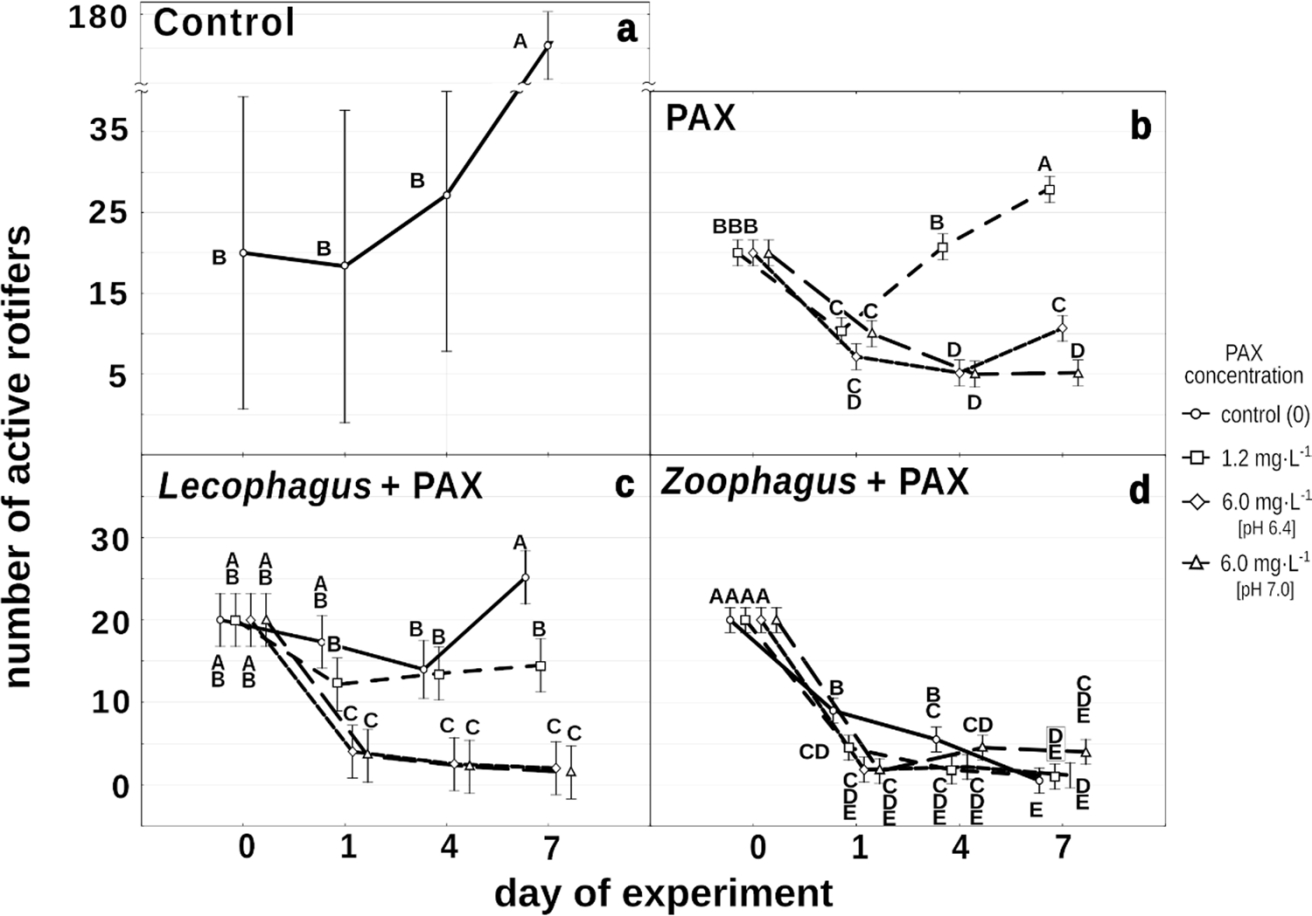
Changes in the mean number of active rotifers in **a** Żywiec® water (control), **b** subjected to different PAX concentrations (PAX), and **c** subjected to simultaneous influence of PAX and *Lecophagus*, and **d** PAX and *Zoophagus*. Points sharing any common letter do not differ significantly. Whiskers indicate 95% confidence intervals

In the control wells, the number of active rotifers was slowly increasing between the 1st and 4th days and by the 7th day had risen significantly to exceed eightfold the initial number (Fig. 1a).

In both PAX solutions containing 6 mg L^−1^ of aluminum ions, the number of active rotifers decreased significantly during the first 24 h and continued to fall slightly over the following 3 days. In the non-normalized solution, it rose slightly, yet significantly on the last day of observations. Apart from this, the differences in the number of active rotifers between normalized PAX with pH = 7.0 and that with pH = 6.4 were not significant (Fig. 1b).

In the PAX solution containing 1.2 mg L^−1^ of aluminum ions, after a short-lasting decline, rotifer numbers had already returned to the initial level by the 4th day and significantly increased at the end of the experiment (Fig. 1b). The differences were significant at F_6,132_ = 55.56, *p* < 0.01.

### Experiment II: the influence of PAX on interactions between early developmental stages of predatory fungi and Lecane rotifers

When rotifers were subjected only to conidia of *Lecophagus*, without PAX addition, their number decreased during the first 4 days, after which it significantly increased, exceeding the initial value (Fig. 1c).

When rotifers were subjected simultaneously to conidia of *Lecophagus* and PAX solutions, their numbers decreased significantly in all treatments within the first 24 h. The most pronounced, almost identical decline was observed in the case of both treatments with a high concentration of aluminum ions. The differences between the treatments were not statistically significant (Fig. 1c). In the case of the low PAX concentration, after the initial decline, the number of rotifers increased, albeit not significantly. The differences between rotifer numbers in the control and the low PAX concentration on the one hand and both high PAX concentrations were significant at F_9,79_ = 8.7603, *p* < 0.01.

A different picture was observed in the treatment with conidia of *Zoophagus*. This fungus appeared very effective in trapping rotifers. Already after 24 h, less than half of the rotifers survived both in control and in all the PAX treatments (Fig. 1d). After a week, only one or two rotifers in any of the control wells (without PAX) remained active. A similar situation was observed for all the PAX treatments, but the fall on the first day of the experiment was significantly bigger than in the control. At the end of the experiment, the highest number of active rotifers was noted in the presence of predatory fungi in the normalized PAX concentration (pH = 7.0), but the difference in comparison to other treatments was not significant. Interestingly, the lowest number of active rotifers on the last day was observed in the control. The difference in the number of rotifers at the beginning of the experiment and on the other days was significant at F_9,79_ = 7.436, *p* < 0.01.

The results of subjecting rotifers simultaneously to PAX and *Lecophagus* were similar to those in the experiment without the fungus, apart from the control. The numbers of active rotifers remaining after 24 h in treatments with conidia of *Lecophagus* and treatment without predatory fungus were similar (Fig. 1b,c). In the absence of PAX, however, *Lecophagus* reduced the number of active rotifers as strongly as in treatments with *Lecophagus* and PAX together (Fig. 1c), whereas in the experiment in which only the influence of PAX was tested, the number of active rotifers in the control was much higher (Fig. 1a).

**Fig. 2:**
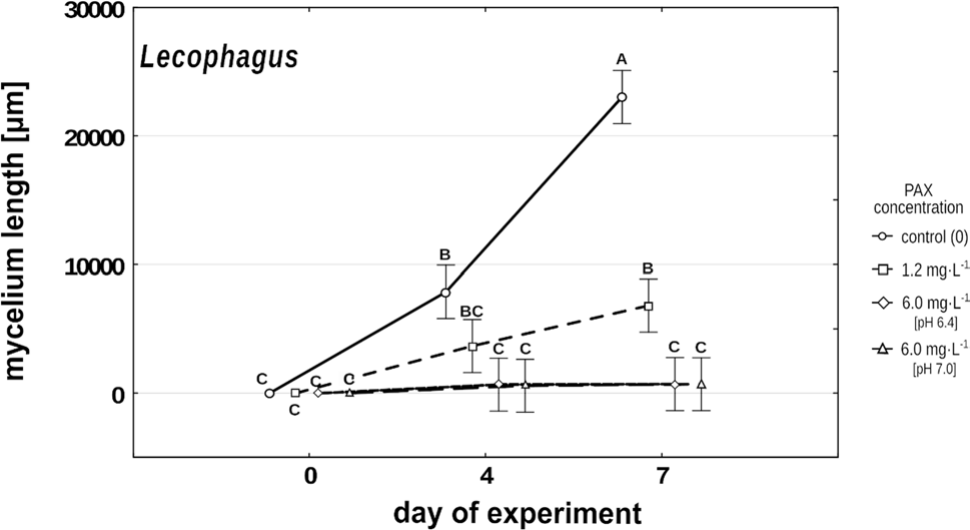
Changes in mean *Lecophagus* mycelium length in control and different PAX concentrations. Points sharing any common letter do not differ significantly. Whiskers indicate 95% confidence intervals

PAX solution clearly affected *Lecophagus* sp. as the growth of its mycelium starting from conidia was drastically limited in the high concentration of PAX solution (Fig. 2). In both high concentrations (normalized and non-normalized), mycelium ceased growing between the 4th and 7th days of the experiment, and after a week, its length did not exceed 180 µm. In the control, in Żywiec® spring water, during this period, the mycelium expanded threefold and on the 7th day was on average almost 30 times longer than in the treatments just discussed. Even the low concentration of aluminum ions still negatively affected the growth of *Lecophagus* mycelium. In this treatment, on the 7th day, the average mycelium was over three times shorter in comparison to the control (Fig. 2). The differences were significant at F_6,60_ = 27.279, *p* < 0.001.

The growth rate of mycelium in spring water, at the low and both high concentrations of aluminum, varied between 0.26 and 0.52/d; 0.11 and 0.26/d; and 0–0.21/d respectively. Also, the formation of new a generation of conidia clearly depended on PAX concentration. New conidia were observed already on the 4th day of the experiment in five out of six control wells, as well in three out of six wells with a low concentration of aluminum. In the two high PAX concentrations, new conidia did not appear until the end of the experiment.

**Fig. 3:**
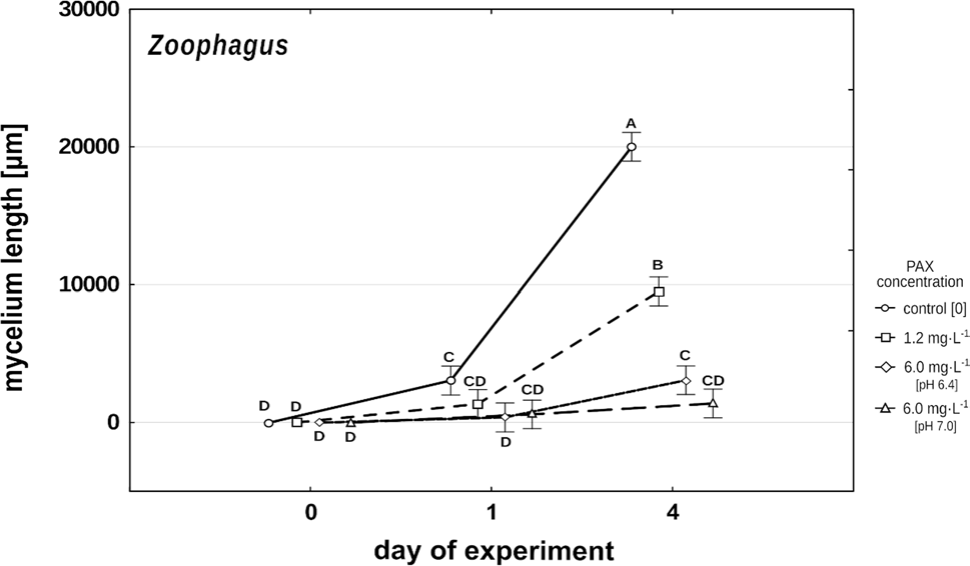
Changes in mean *Zoophagus* mycelium length in control and different PAX concentrations. Points sharing any common letter do not differ significantly. Whiskers indicate 95% confidence intervals

The mycelium of *Zoophagus* in Żywiec® spring water grew very rapidly, and already after 4 days, it was at least twice as long in comparison to mycelium treated with a low concentration of PAX (Fig. 3). In the high concentrations of PAX solution (normalized and acidic), the growth of the mycelium was strongly limited. Its length after 4 days was significantly lower in comparison to that in the low concentration (F_6,60_ = 76.550, *p* < 0.001) (Fig. 3). It was often the case in the high PAX concentration that even if a conidium germinated and mycelium started to grow, it tended to degenerate. In the low PAX concentration, the fungus grew significantly faster than in the high concentration but slower than in control wells. Both differences were significant at F_6,60_ = 76.550, *p* < 0.001. In Żywiec® spring water, the mycelium grew so rapidly that already after 4 days we were not able to measure all the mycelium as it exceeded the field of view, reaching a length over 20 000 µm. For data analysis, we decided to assign a length of 20 000 µm to these cases.

### Experiment III: the effect of PAX on the trapping efficiency of developed mycelium

**Fig. 4:**
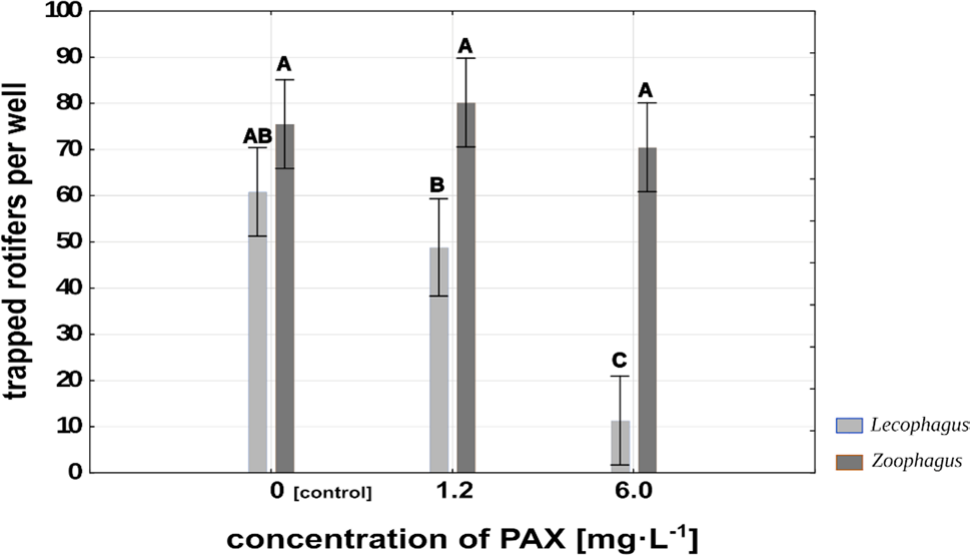
Mean number of rotifers trapped by the fungi during 3 h in control and two different PAX treatments. Points sharing any common letter do not differ significantly. Whiskers indicate 95% confidence intervals

The mean number of rotifers caught by well-developed mycelium clearly depended on the PAX concentration in the medium. *Lecophagus* mycelium subjected to the influence of high PAX concentration had trapped on average 10 rotifers per well during 3 h after the substitution of PAX solution for fresh spring water. Mycelia earlier treated with a low concentration of aluminum salts, and these remaining in spring water were much more effective and caught on average 49 and 61 rotifers per well respectively (Fig. 4). The differences between high PAX concentration and both control and low PAX concentration were significant at F_2,29_ = 11.45, *p* < 0.001, whereas the differences between control and low PAX concentration were not significant.

*Zoophagus* seemed to be less sensitive to the influence of PAX. The mean number of rotifers caught by its developed mycelium during 3 h after the exchange of the medium for fresh Żywiec® water was apparently independent of the aluminum ion concentration to which the fungus was earlier exposed (Fig. 4). In all treatments, the mycelium was very effective in trapping rotifers. On average, it caught between 70 and 80 rotifers per well, and the differences were not significant.

**Fig. 5:**
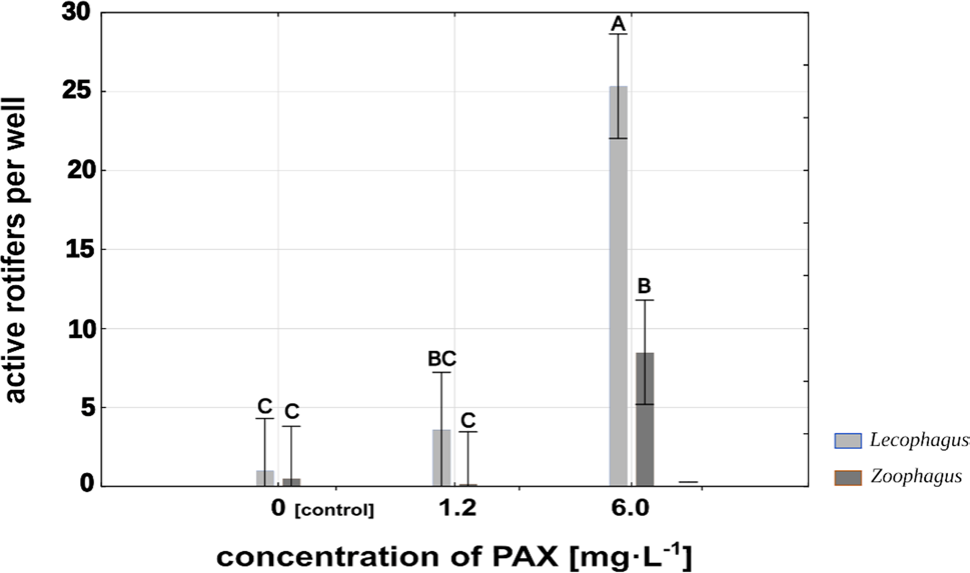
Mean number of active rotifers remaining 24 h since the release into the wells in control and two different PAX treatments. Points sharing any common letter do not differ significantly. Whiskers indicate 95% confidence intervals

After 24 h, the number of active rotifers was the highest in the presence of developed *Lecophagus* mycelium in the high concentration of aluminum ions (Fig. 5), fivefold exceeding that in the low PAX concentration (F_2,29_ = 14.409, *p* < 0.001). In the absence of aluminum ions, only one or two rotifers survived for 24 h, but the difference between the control and the low PAX concentration was not significant (Fig. 5).

The results were very similar for *Zoophagus* mycelium. After 24 h, the highest number of active rotifers per well was noted in the treatment with a high concentration of aluminum ions (Fig. 5), and it was significantly different from the other treatments at F_2,29_ = 14.409, *p* < 0.001. Almost no rotifers survived in the other treatments, and their numbers did not differ significantly from those in the *Lecophagus* treatments.

### Experiment IV: conidia resistance to PAX and starvation

**Fig. 6:**
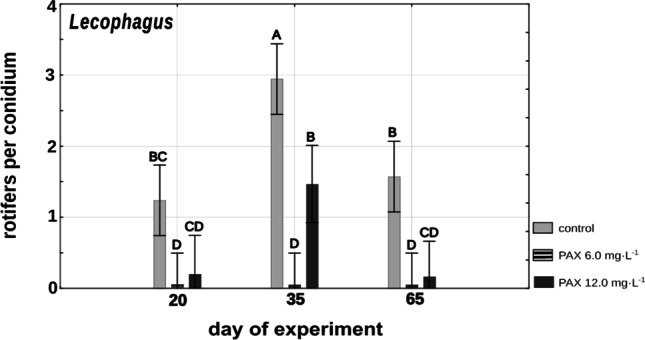
Mean numbers of rotifers trapped by *Lecophagus*, after 3, 5, and 9 weeks of remaining under different PAX concentrations and Żywiec water. Points sharing any common letter do not differ significantly. Whiskers indicate 95% confidence intervals

High concentration of PAX combined with the absence of a food source led to the total inability of *Lecophagus* conidia to trap rotifers when provided again. After 3 weeks under the influence of PAX, none of the conidia managed to trap rotifers in 3 h after rinsing in spring water and being provided with rotifers (Fig. 6). Conidia in the treatment with a low concentration of polyaluminum chloride trapped no more than one rotifer each regardless of the time. A slightly higher number of rotifers were trapped by *Lecophagus* conidia in control wells, but still, the numbers were very low. Interestingly, after 5 weeks, the conidia in the control wells and those treated with low PAX concentration were significantly (F_4,43_ = 3.871, *p* < 0.001) more successful in catching rotifers than after 3 or 9 weeks (Fig. 6).

**Fig. 7:**
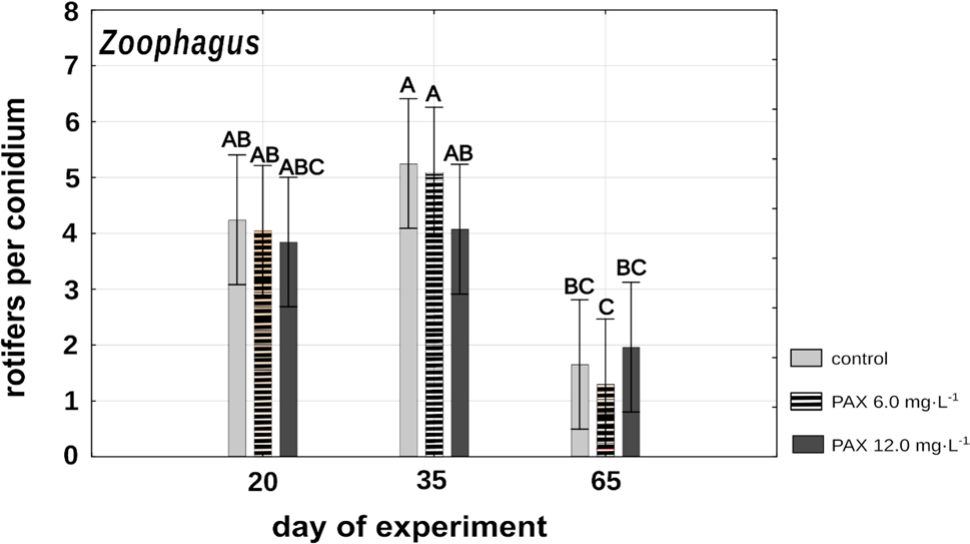
Mean numbers of rotifers trapped by *Zoophagus* after 3, 5, and 9 weeks of remaining under different PAX concentrations and Żywiec water. Points sharing any common letter do not differ significantly. Whiskers indicate 95% confidence intervals

The ability of *Zoophagus* conidia to catch rotifers appeared much less sensitive to aluminum ions. The fungi remaining under the influence of PAX solutions as well as those in the control after 3 and 5 weeks were trapping rotifers in numbers not differing significantly (Fig. 7). Only after 9 weeks did the efficiency of catching rotifers fall considerably, but again there was no difference between treatments and control (Fig. 7). After 3 and 5 weeks of remaining under the influence of aluminum ions, four rotifers were trapped by a conidium on average, independently of PAX concentration in the medium. The lowest number of rotifers trapped by the fungus was noted in all treatments after 9 weeks of the experiment when on average only 1–2 rotifers were caught per conidium.

## Discussion

Even though PAX is widely applied in WWTPs to limit the proliferation of troublesome filamentous bacteria, as well as in freshwater lakes for removal of cyanobacteria and/or phosphorus, its impact on microfauna has not been thoroughly studied. Earlier research has concentrated on the influence of polyaluminum salts on *Daphnia* sp. (Piasecki and Zacharzewski 2010, Jančula et al. 2011) showing a negative impact of PAX on its growth. Hansen and Schuurman (1999) mentioned that polyaluminum chloride had a negative effect on the microfauna of activated sludge only at extremely high concentrations. However, the authors used other PAX coagulants, and they did not mention the species of observed ciliates and rotifers. As our earlier research showed that *L. inermis* rotifers are important components of activated sludge due to their ability to limit filamentous bacteria (Fiałkowska and Pajdak-Stós 2008; Kocerba-Soroka et al. 2013; Drzewicki et al. 2015), it is vital to characterize their tolerance to chemicals applied in WWTPs.

### Effects of PAX concentration on rotifers

Our current experiment on the effect of polyaluminum chloride on rotifers showed that *L. inermis* (clone 1.A2.15) are sensitive to PAX in concentrations reflecting dosing of the coagulant at the range between 2 and 5.5 g L^−1^ MLSS, as applied in wastewater treatment plants. In a high concentration of aluminum ions (6 mg Al^3+^ L^−1^), after a short-lasting decline, rotifers were able to survive for a week, but they did not proliferate. Rotifers exposed to low PAX concentration (1.2 mg Al^3+^ L^−1^) not only survived to the end of the trial but their number even increased (Fig. 1). Apparently, clone 1.A2.15 is more resistant to aluminum ions than the clones of *L. inermis* used in earlier toxicological tests (Klimek et al. 2013). In that earlier research, the mean EC50 value calculated for the 24-h mortality test conducted with Lk1 and Lk3 clones was as low as 0.012 mg Al^3+^ L^−1^ (Klimek et al. 2013). However, it is worth underlining that in those experiments, freshly hatched rotifers (< 1 day) were used. One can expect that juveniles are more sensitive to toxins than adult forms. In our experiment, rotifers were randomly picked from a well-established culture, and in this way, their age distribution reflected the situation in a real WWTP. Moreover, in earlier research, simple aluminum salts such as AlCl_3_ and Al_2_(SO_4_)_3_ were used. PAX-18 is an aqueous solution of polyaluminum chloride. It is possible that aluminum ions in such solutions are less reactive than those originating from the salts mentioned above. In comparison, the 48-h acute toxicity test conducted using AlCl_3_ on neonates of *Lecane quadridentata* found that LC50 value for Al ions equaled 0.5 mg L^−1^ (Guzman et al. 2010). Although rotifers of *L. inermis* are able to survive in PAX concentration reflecting ranges applied in WWTPs, their growth rate is very low and thus could not keep pace with the loss of individuals removed from WWTP with excessive sludge. The inhibition of growth of *L. inermis* exposed to polyaluminum chloride is clearly visible when we compare the number of rotifers in PAX treatment with control without aluminum ions, where the rotifers proliferated rapidly, and at the end of the experiment, their number was eight times higher than in PAX treatment (Fig. 1).

### Effects of predatory fungi on rotifers

Rotifers inhabiting activated sludge are also endangered by predacious fungi (Pajdak-Stós et al. 2016; Fiałkowska and Pajdak-Stós 2018). Our experiments showed that rotifers subjected simultaneously to PAX solution and conidia of *Lecophagus* sp. had an even lower chance of survival than in the absence of the fungus. In the high concentration of polyaluminum chloride, only one or two rotifers per well survived for a week (Fig. 1). Also in the low concentration of PAX and in Żywiec® spring water, the rotifers’ growth was strongly limited. Even though they survived to the end of the trial, their numbers remained at the same level. The comparison of the number of active rotifers in Żywiec® without (Fig. 1a) and with (Fig. 1c) *Lecophagus* shows how strongly the fungus alone can reduce rotifer numbers. Approximately eight times fewer rotifers remained in wells inoculated with *Lecophagus* in comparison to controls with neither PAX nor conidia (Fig. 1c).

The other fungus, *Zoophagus*, appeared to have an even stronger negative effect on the rotifers. In the presence of its conidia and then mycelium, the number of rotifers drastically declined, and at the end of the experiment, only solitary rotifers remained in low PAX concentration and Żywiec® spring water, whereas the highest number of active rotifers remained in the high concentration of PAX (pH 6.4). The observed differences, however, were not significant (Fig. 1d). This result could suggest that *Zoophagus* effectiveness in trapping rotifers was somehow inhibited by aluminum ions, but still, rotifers’ chances of survival were rather low. This suggests that because polyaluminum salts have such a strong negative effect on *Lecane*, the simultaneous application of rotifers and PAX to control bulking is not justified.

### Effects of PAX concentration on the growth of fungi

Looking at the effect of aluminum ions on the growth of the fungus, we can see that polyaluminum chloride has also a pronounced negative effect on the development of mycelium in the case of both investigated predatory fungi. The pattern of the dependence of growth of *Lecophagus* sp. and *Zoophagus* sp. mycelium on PAX concentration is similar (Figs. 2 and 3). When subjected to the high concentration of aluminum ions, the growth of mycelia is almost entirely restricted. *Lecophagus* sp. grew almost four times faster in control conditions in comparison to the low concentration of aluminum ions (Fig. 2). Similarly, *Zoophagus* grew much faster in control than in the PAX treatments (Fig. 3).

### Effects of PAX concentration on the relationships between rotifers and fungi

One might argue that inhibited growth of mycelium in aluminum ion solutions is an indirect effect of lower rotifer availability at high concentrations of PAX. To test this, we checked whether and to what extent developed mycelium is impaired by being subjected to different concentrations of aluminum ions when rotifers are absent. Such an experiment simulates the situation where PAX solution is applied first in order to prevent bulking of activated sludge and then the rotifers are added. In the case of *Lecophagus* sp., very few rotifers were caught by the fungus previously subjected to the influence of high PAX concentration. This result is promising in the context of limiting fungal development in a real scale WWTP; reduction of fungus growth by PAX would increase rotifers’ chances to survive (Fig. 4). In research conducted in a real scale WWTP, the unexpected appearance of predatory fungi following application of rotifers brought about a rapid decline in rotifer density (Pajdak-Stós et al. 2017a, b). At the time of this experiment, no coagulant had been used to overcome sludge bulking, so it cannot be ruled out that if PAX had been used prior to introduction of the rotifers, it might have prevented rapid growth of predatory fungi.

The situation was slightly different in the case of *Zoophagus*. No difference was observed in the number of rotifers trapped by mycelium earlier treated with PAX solutions in comparison to the spring water. Nevertheless, similar to the experiment with *Lecophagus*, far more rotifers survived for 24 h in the presence of mycelium earlier subjected to a high concentration of aluminum ions in comparison to control and low PAX concentration (Fig. 5).

### Effects of PAX concentration on conidia

The most pronounced differences between the two fungi were observed in the activity of conidia that remained under the influence of the high concentration of polyaluminum chloride for a prolonged period. In such conditions, conidia of *Lecophagus* lost the ability to trap rotifers after just 3 weeks, whereas in the case of *Zoophagus*, there were no differences between treatments and control in the number of rotifers caught (Figs. 6 and 7), and this tendency was observed throughout the whole experiment. Differences in the vulnerability of predatory fungi to aluminum ions could be related to the permeability of membranes and/or cell walls. In experiments conducted on four fungal potato pathogens belonging to Ascomycota and Oomycota, Avis and co-authors showed that aluminum chloride antifungal activity consists in its negative influence on membrane integrity (Avis et al. 2007). As *Lecophagus* also belongs to Ascomycota, it seems plausible that its membranes may be sensitive to PAX solution. Furthermore, *Lecophagus*, unlike *Zoophagus*, is not an obligatory predator and can utilize nutrients dissolved in the medium. Apparently, in the case of *Lecophagus*, there might not be a sufficient barrier for toxins, especially when no rotifers are available, and the fungus is forced to switch to soluble food.

Interestingly, the experiment showed surprising resistance of the fungi to adverse environmental conditions; conidia of both *Lecophagus* and *Zoophagus* maintained their ability to catch rotifers even after 3 months of starvation. *Lecophagus* conidia trapped more rotifers in control conditions in comparison to those subjected to PAX, whereas in the case of *Zoophagus*, there were no differences between treatments and control, although the number of trapped rotifers after three months was less than half the number seen in the trial lasting 1 month (Fig. 7).

### Ecological importance of the awareness of the relationship between rotifers and predatory fungi in activated sludge treated with PAX

Fiałkowska and Pajdak-Stós (2008) pointed out the negative correlation between number of rotifers and filamentous bacteria in a few WWTPs. This was confirmed by observations conducted in a WWTP treating brewery influent, where the rapid decrease in the density of type 021 N was clearly correlated with the increase in the number of rotifers (Kocerba-Soroka et al. 2013). Our further experiments conducted in a full-scale WWTP showed that application of rotifers resulted in considerable increase in their number leading to significant limitation of the two most troublesome filamentous organisms: *M. parvicella* and Actinomycetes*.* Sudden appearance of predatory fungi in amounts high enough to be observed in microscopic slides resulted in a dramatic drop in the number of rotifers followed by the revival of Actinomycetes (Pajdak-Stós et al. 2017a). Apparently, PAX solution that is applied to control activated sludge bulking by *M. parvicella* is also suitable for mitigation of predatory fungi which endanger populations of beneficial rotifers. As *Lecophagus* seems to be more sensitive to PAX, the application of the latter is especially reasonable in the situation when this fungus threatens the success of employing rotifers. As the population of *Lecane* is also endangered by aluminum ions, the application of PAX to control predatory fungi should take place before the inoculation of rotifers used for bulking control with an interval of at least a few days between those two treatments. Such a procedure would protect rotifers in two ways: PAX will limit the growth of predacious fungi, whereas the concentration of polyaluminum chloride will sufficiently decrease to levels safer for the rotifers. Potentially, the sooner the rotifers are applied, the lower the risk of recontamination of the activated sludge with predatory fungi present in incoming influent. The shift in time of the application of chemical and biological methods should lead to the optimization of filamentous bacteria control. Such a procedure could result in significant limitation of the aluminum ions entering waterbodies and in this way minimize the toxicological risk not only for the rotifers but for the other aquatic organisms and indirectly for humans.

## Supplementary Information

Below is the link to the electronic supplementary material.

Supplementary file1 (TXT 2 KB)

## Data Availability

All data are available on request from corresponding author (edyta.fialkowska@uj.edu.pl). *Lecophagus* sp. Z1 is available from USDA-ARS as strain ARSEF 14203. SSU sequences are deposited at NCBI GenBank under accessions MZ326345 and OK156471.
